# 

**Published:** 1861-10

**Authors:** 


### WORKS PUBLISHED DURING THE QUARTER.

### 1. *Medicine.*

BENNET.—Mentone and the Riviera as a Winter Residence. By J. Henry Bennet, M.D. Post 8vo, cloth, 3s. 6d.

BRINTON.—On the Medical Selection of Lives for Assurance. By W. Brinton, M.D. Third Edition, 12mo, cloth, 2s.

CHAVASSE.—Advice to a Mother on the Management of her Offspring. By Pye Henry Chavasse, F.R.C.S. Sixth Edition, fcap. 8vo, 2s. 6d.

HALL.—Biographical Memoirs of Marshall Hall, M.D., F.R.S., Corresponding Member of the Institute of France, and Foreign Associate of the Academy of Paris. By his Widow. With Portrait. 8vo, cloth, 14s.

LEE.—Homburg and Nanheim. By Edwin Lee, M.D. Second Edition. Post 8vo, 1s.

M'NICOLL.—A Handbook for Southport, Medical and General: with Copious Notices of the Natural History of the District. By David H. M'Nicoll, M.D., M.R.C.P. Second Edition, post 8vo, cloth, 3s. 6d.

MOORE.—Memorandums and Recollections on Gout and Rheumatism, and their Treatment. With a few Practical Remarks on Sciatica and Lumbago. By Edward Duke Moore, L.R.C.P.E. 8vo, 1s.

PRIDHAM.—On Asthma: the Result of Treatment of nearly One Hundred Cases. By T. L. Pridham, M.R.C.S. M.S.A. 8vo, price 2s.

SMITH.—Health and Disease as influenced by the Daily, Seasonal, and other Cyclical Changes in the Human System. By Edward Smith, M.D., F.R.S. Small 8vo, cloth, 10s. 6d.

TAYLOR.—A Manual of Medical Jurisprudence. By Alfred S. Taylor, M.D., F.R.S. Seventh Edition, Revised and Enlarged, fcap. 8vo, cloth, 12s. 6d.

TAYLOR.—The Climate of Pau; with a Description of the Watering-Places of the Pyrenees, and of the Virtues of their respective Mineral Sources in Disease. By Alexander Taylor, M.D., F.R.S.E. Third Edition, considerably Altered, post 8vo, cloth, 7s.

### 2. *Surgery.*

DUNCAN.—The Method of Drill, the Gymnastic Exercises, and the Manner of Teaching Speaking, used at Essex Hall, Colchester, for Idiots, Simpletons, and Feeble-Minded Children. By P. Martin Duncan, M.B. Lond. Fcap. 8vo, cloth, 2s.

HOLMES.—A System of Surgery, Theoretical and Practical, in Treatises by Various Authors, Arranged and Edited by T. Holmes, M.A. Cantab., Assistant-Surgeon to the Hospital for Sick Children. Vol. II.—Local Injuries—Diseases of the Eye. 8vo, cloth, 1*l.* 1s.

HUMPHRY.—The Human Foot and the Human Hand. By G. M. Humphry, M.D., F.R.S. 12mo, cloth, 4s. 6d.

TRUMAN.—The Necessity of Plasticity in Mechanical Dentistry. Read before the Odontological Society. By Edwin Truman, M.R.C.S. 8vo, 1s. 6d.

WALTON.—A Treatise on the Surgical Diseases of the Eye. By Haynes Walton. Second Edition, Re-written, with 173 Illustrations, 8vo, cloth, 14s.

### 3. *Chemistry.*

BEASLEY.—The Druggist's General Receipt-Book: comprising a Copious Veterinary Formulary and Table of Veterinary Materia Medica; Patent and Proprietary Medicines; Druggists' Nostrums, &c. By Henry Beasley. Fifth Edition, Revised, 18mo, cloth, 6s.

BOWMAN.—Bowman's Practical Chemistry, including Analysis. With numerous Illustrations on Wood. Fourth Edition. Edited by C. L. Bloxam. Fcap. 8vo, cloth, 6s. 6d.

RUSSELL.—The Tannin Process in Photography. By C. Russell. 12mo, cloth, 2s.

---

### FOREIGN MEDICO-PSYCHOLOGICAL LITERATURE.

### INDEX TO VOL. I.

Akbare Jubabut (Native Indian Journal), 155
American medical men, conduct of, in the war, 691
Apothecaries' Company *v.* College of Physicians, 478
Arcanum, the Great, 368
Asylums, cottage, 213
——— middle class, xxxvii

Boismont, Brierre de, on the medico-legal relations of hallucinations, 668
Brodie, Sir Benj., eyesight of, 322
Baillarger, Dr., on hypochondriacal insanity as a precursor of general paralysis, 170
——— on the responsibility of epileptics, 507
Baly, Dr. William (In Memoriam), 334
Bath, the Turkish, 367
Belloc, Dr., on the legal responsibility of the insane, 346
Berthier, on fever in its relations to insanity, 343
Bibliography, medical, 323
Brain, on cysticercus cellulosæ in the, 515
Bray, Mr. C., on the education of the feelings, 330
Book-hawking union, xii
Books: quarterly lists of books published, 331, 505, 711
Brown, Mr. I. Baker, on surgical diseases of women, 488
Browne, Dr. W. A. F., on cottage asylums, 213
——— Mr. J. Crichton, on clinical psychology, 707

Cafés, insalubrity of atmosphere of, 518
Camp, Maxime du, *Mémoires d'un Suicide*, 568
Castelnau, Dr., on interdiction, 285
Cavour, Count, death of, 485
Chance's, Dr., translation of Virchow's cellular pathology, 311
China, health of troops in, 155
Christison, Dr., on some of the medico-legal relations of intemperance, 500
Convict rule and feeding, xxxviii
Copland, Dr., on consumption and bronchitis, 325
Cormac, Dr. Mc, a plea for the insane, 262
Cottages, labourers', 155
Cottage asylums, 213, 449
Cretinism, on the educational treatment of, 626

Dale, Mr. W., on the present state of the medical profession, 504
Diphtheria, 25
Doctor? who is a, 394
Drainage of the kingdom, xvii

Eccentricity, moral phenomena of, 173
Egyptian frigate, case of, at Liverpool, 552
Eisenach, meeting of German alienist physicians at, in 1860, 156.
England, state of lunacy in, 642
Entozoa in the insane, particularly oxyuris vermicularis, 158
Epidemiological society, transactions of, 500
Epidemic diseases, spread of, 205
Epileptics, the responsibility of, 507
Epileptiform convulsion, case of, 723

Fairless, Dr., on construction of asylums, 710
Female physicians, 586
Fevers, remarks on causation of, 552
Figuier, *Histoire du Merveilleux*, 1
Flourens, *De la Raison, du Génie, et la Folie*, 133
Freke, Dr. on origin of species by organic affinity, 327

Gheel: the gheel question, by Dr. Mundy, 399
Goodfellow, Dr., on Bright's disease and dropsy, 700
Gossip, medical, 150, 319, 478, 682

Gossip, literary, and record, 323, 486, 692
Guardia, Dr. on the study of insanity, 697
Guy, Dr., on forensic medicine, 330

Hallucinations, Brierre de Boismont on medico-legal relations of, 668
Harris, Rev. T. L., *Arcana of Christianity*, 1
Harvey, neglected state of burial-place of, 153
Herschel, Sir John, on Meteorology and Physical Geography, 701
Hood, Dr., on criminal lunatics, 31
Homœopaths, consultations with, 483
Honoraria, 275
Hospitals, special, 271
——— term of office in, 323
Hulme's, Mr. F., translation of Moquin-Tandon's *Elements of Medical Zoology*, 489
Hunter, John, recovery and publication of posthumous manuscripts of, 319
Hyperæsthesia, on mental, 512
Hysteria and hysteromania, 168

Ingleby, Dr., 357
Insane, entozoa in, 158
——— hæmatic swelling of ears in, 159
——— on the legal responsibility of, 346
——— moral responsibility of, 712
——— a plea for the, 262
Ireland, district lunatic asylums of, discussion in Parliament on, 690
Insanity, on confounding of persons as a symptom of, 341
——— on fever in relation to, 343
——— a form of, known as congestive mania, 160
——— hypochondriacal, as a precursor to general paralysis, 170
——— supposed increase of, in England, 643
——— moral phenomena of, and eccentricity, 174
——— lucid, 659
——— on opinion in the treatment of, 342
——— osteomalacia as a cause of, 158
——— statistics of, 716
——— the philosophy of, by a quondam lunatic, 327
——— the *Times* on the plea of, xix
——— unrecognised, 659
Interdiction, on, 25

Joire, Dr., organic lesion of certain nervous centres, 516
Jones, Dr. Griffith, case of, 689
Judgments, on the distinction between analytical and synthetical, 357

Latham, Dr. R. G., on the syllogism, 37
——— on the structure of the inductive syllogism, 253
Lectures, introductory, session 1860-61, 152
——— Mr. Milton's at the College of Surgeons, 152
——— Lettsomian, 153
——— at London College of Physicians, 153
Legislation, physiological essay on, 285
Literature, Foreign, medico-psychological, 156
Linas, Dr., on delirium as a sign of general paralysis, 348
Liverpool, case of Egyptian frigate at, 552
Lunacy, regulation bill, xxix
——— state of, in Scotland, 416
——— state of, in England, 642
——— 15th report of English, 642
——— 3rd report of Scottish, 416
——— supposed increase of, in England, 643
Lunatics, criminal, 31
——— on "non-restraint," and the increase of, 124
——— child-stealing by a, xi
——— asylums, district, discussion in Parliament, 690
——— murderers, 679

Mania, congestive, 160
——— and hysteromania, 158
Marvellous, the, 1
Maternal love in nature, by J. L. C. Schroeder van der Kolk, professor in the University of Utrecht, 103
Mayo, Dr. Thomas, on the moral phenomena of insanity and eccentricity, 173
Meadows, Dr., on errors of homœopathy, 710
Medical Act, hitch in, 687
——— defect in penal clause of, 688
Medical education, new regulations on, 684
——— gossip, 150, 319, 478, 682
——— registration, 81
——— societies, proposed amalgamation of, 481
——— students, 636
Medicine, the study of, 537
Metanoia; a plea for the insane, 262

Meyer, Dr., on opium in the treatment of insanity, 342
Millar's, Mr., *Hints on Insanity*, 329
Miller's, J., *Nephalism*, 504
Milroy, Dr. Gavin, on quarantine and the spread of epidemic diseases, 205
——— a case of Egyptian frigate at Liverpool, with remarks on causation of fevers, 552
Militia surgeons, 155
Miscellanea Medica, 293, 444
Moquin-Tandon, elements of medical zoology, 489
Morals, the *Times* on legislative interference in, ix
Morel, Dr., on Non-restraint, 124
Mortality in town and country districts, xxv
——— influence of temperature on mortality, xxxv
Mundy, Dr., the Gheel question, 399
——— on educational treatment of cretinism, 626
Munk, Dr., Roll of College of Physicians, 693
Murders by unrecognised lunatics, xlviii—liv

Nervous centres, on an organic lesion of certain, 516

Ordronaux, Dr., on insanity, 512
Orientalism, 603
Osteomaldacia, as a cause of insanity, 158
Oxyuris vermicularis in the insane, 158

Paralysis, general, medico-legal studies on, 60
——— hypochondriacal insanity as a precursor of, 170
——— a special form of delirium in, 345
——— on delirium as a sign of, 348
Parsees, exclusion of from medical service of army and navy, 690
Pathology, cellular, 311
Physic, art of rising in, 303
Physical forces, the correlation of the moral and, vii
Physicians, London College of, (new order of licentiates), 150
——— Roll of, 693
——— *v.* Apothecaries Company, 478
Physicians, Edinburgh College and the War Office, 151
Physicians, female, 586
Physicians, German alienist, meeting of, at Eisenach in 1860, 156
Plague, a rare tract on, 293
Population, displacement of in large towns, xxvi

Quackery, restraint of, 688
Quarantine and the spread of epidemic diseases, 205

Radcliffe, Dr. C. B., on epileptic and other convulsive affections of the nervous system, 493
Reason, genius, and madness, 133
Ricord, retirement of, 154
Road murder, psychologically considered, 424
Routh, Dr., on infant feeding and mortality, 325

Saulle, Dr. Legrand de, on special forms of delirium in general paralysis, 345
——— Dr. L. D. on the insalubrity of the atmosphere of cafés, 518
Scotland, Registrar-General's report, xxxi
——— illegitimacy in, xxxii
——— comparative instruction of England and, xxxiv
——— the state of lunacy in, 416
——— medical profession of, on the Lunacy Act, 519
Shaw, Mr. A., on Sir C. Bell's dis coveries, 330
Sieveking, Dr., on epilepsy, 496
Skeyne, Dr. Gilbert, Medicinar to James IV., 293
Smith, Dr. E., on cyclical changes in the human being, 703
——— Dr. J. E., aural diagnostics, 498
——— Dr. Pyemont, statistics of insanity, 710
——— on cysticercus cellulosæ in the brain, 515
Snell, Dr., on a symptom of insanity, 341
Social Science Association at Glasgow, 155
Soliloquy, medical, 412
Spanish medical practice 16th century, 300
Specialists and Specialities, 54
Spiritual Magazine, the, 1
Statistical Congress, 378
Statistics, vital, of 1860, xxiv
——— the amenities of, 378
——— of insanity, 655
Stepney murderer (Mullens), xix
Superstition and Crime, xliv
Surgeons, Royal College, list and balance-sheet of, for 1860, 154
Surgery, modern, 239
Suicide, the classic land of, 183

Suicide, remarkable example of, xliii
——— statistics of, at Turin, 1855-59, 346
——— æsthetics of, 563
Swedenborg's dreams, 612
Syllogism, on the structure of the inductive, 253

Taylor, Dr. A. S., medical jurisprudence, 703
Trelat, Dr., sur folie lucide, 659
Titles, Medical, 152
Tobacco controversy, the *Daily Telegraph* on the, v
——— smoking, ii
——— physical evils of, iii
——— physical benefits of, v
Todd, Robert Bentley, (In memoriam), 143
Torchio, Mr. F., on suicide at Turin, 1855-59, 346

Unrecognised insanity, 659, xlviii—liv

Vacation, the, 349
Virchow, on cellular pathology, 311

Walton Haynes, on the surgical diseases of the eye, 694
Walworth murderer, Youngman, xx
Webster, Dr. J., on the leper hospital at Granada, 704
Whewell, Dr., the Platonic dialogues, 702
Worthington, Dr., on congestive mania, 160

Year-Book of Medicine and Surgery, 707

END OF VOL I.

54, Princes Street, Leicester Square,

London, W.

*1st October*, 1861.

### JOHN W. DAVIES

*BEGS LEAVE TO ANNOUNCE THE FOLLOWING*

### LIST OF NEW WORKS

*to be published by him during the* Medical Session, 1861-2.

---

### A COMPENDIUM OF CHEMISTRY,

For the Use of Students.

BY WILLIAM THOMAS BRANDE, D.C.L., F.R.S.L. & E.,
Of Her Majesty's Mint, Honorary Professor of Chemistry in the Royal Institution, Fellow of the University of London ;

AND ALFRED S. TAYLOR, M.D., F.R.S.,
Fellow of the Royal College of Physicians, and Lecturer on Chemistry and Medical Jurisprudence in Guy's Hospital.

---

### ON THE ARCUS SENILIS;

OR,

**FATTY DEGENERATION OF THE CORNEA.**

WITH NUMEROUS ILLUSTRATIONS.

BY EDWIN CANTON, F.R.C.S.,
Surgeon to the Charing Cross Hospital.

---

### EVIDENCES OF THE ARRESTMENT OF PHTHISIS,

WITH OBSERVATIONS
ON TUBERCLE AND TUBERCULAR EXPECTORATION,

BEING THE LETTSOMIAN LECTURES DELIVERED BEFORE THE MEDICAL SOCIETY OF LONDON IN 1857.

BY ANDREW CLARK, M.D., F.R.C.P.,
London Hospital, and Emeritus Assistant Physician to the City of London Hospital for Diseases of the Chest.

### TRANSACTIONS

OF THE

### EPIDEMIOLOGICAL SOCIETY OF LONDON.

Vol. I.

---

### SPINAL DEBILITY,

**ITS PREVENTION, PATHOLOGY, AND CURE,**

IN RELATION TO

CURVATURES, PARALYSIS, EPILEPSY, AND VARIOUS DEFORMITIES.

BY EDWARD W. TUSON, F.R.C.S.,

Formerly Surgeon to the Middlesex Hospital.

---

### THE ANATOMY AND PHYSIOLOGY OF THE TEETH.

BY H. T. KEMPTON, M.C.D. Eng., F.L.S.,

Surgeon Dentist to the Royal Infirmary for Diseases of the Ear.

---

### PRACTICAL MEDICAL PRECEPTS FOR PLAIN PEOPLE.

BY ALFRED FLEISCHMANN, M.R.C.S.

---

### THE MEDICAL CRITIC

AND

### PSYCHOLOGICAL JOURNAL.

EDITED BY FORBES WINSLOW, M.D., D.C.L., Oxon.

No. 4, for October.

---

### THE DENTAL REVIEW,

**A MONTHLY JOURNAL OF DENTAL SCIENCE.**

No. 10, Vol. 3, for October.

---

### BIBLIOGRAPHIA MEDICA.

A general List of Works on Medicine and the allied Sciences, including Pamphlets, published during the Session 1860-61.

*Gratis on application.*

**WORKS PUBLISHED BY JOHN W. DAVIES.**

### OBSCURE DISEASES OF THE BRAIN,

AND

**DISORDERS OF THE MIND.**

By FORBES WINSLOW, M.D., D.C.L. Oxon.

Second and Revised Edition, 8vo, cloth, price 16s.

### ON SURGICAL DISEASES OF WOMEN.

By I. BAKER BROWN, F.R.C.S.,

Senior Surgeon to the London Surgical Home for Diseases of Women.

Second Edition, enlarged, with Engravings, 8vo, cloth, 15s.

### STRICTURE OF THE URETHRA;

Its Nature and Treatment in the more Severe Forms;

WITH THE DESCRIPTION OF A NEW GAUGE FOR CATHETERS.

By HENRY SMITH, F.R.C.S.,

Assistant-Surgeon to King's College Hospital.

8vo, sewed, price 1s.

### ON INSUFFICIENCY OF THE AORTIC VALVES IN CONNEXION WITH SUDDEN DEATH;

**WITH NOTES HISTORICAL AND CRITICAL.**

By JOHN COCKLE, M.D., F.L.S.

Physician to the Royal Free Hospital.

8vo, sewed, price 1s.

### INSTRUCTIONS TO MOTHERS AND NURSES IN THE LYING-IN CHAMBER.

By J. C. LORY MARSH, M.D., M.R.C.P. Lond.

Fcap. sewed, price 6d.

**NEW WORK ON PHOTOGRAPHY.**

### THE TANNIN PROCESS.

By C. RUSSELL.

Fcap. cloth, price 2s.

ON THE

### SCOPE, TENDENCY, AND EDUCATIONAL VALUE

### OF THE NATURAL HISTORY SCIENCES:

A DISCOURSE DELIVERED IN THE THEATRE OF THE ROYAL INSTITUTION OF GREAT BRITAIN.

By T. SPENCER COBBOLD, M.D., F.L.S.,

Lecturer on Botany, Zoology, and Comparative Anatomy, at the Medical College, Middlesex Hospital, &c. &c.

8vo, sewed, price 1s.

---

### ON SCARLATINA AND ITS TREATMENT.

By I. BAKER BROWN, F.R.C.S.,

Senior Surgeon to the London Surgical Home for Diseases of Women.

Fcap. cloth, price 3s.

---

A TREATISE UPON

### PENETRATING WOUNDS OF THE CHEST.

FOUNDED UPON ACTUAL OBSERVATIONS IN THE CAMP GENERAL HOSPITAL BEFORE SEBASTOPOL.

By PATRICK FRASER, M.D.,

Late Staff Physician to the Army in the Crimea; Physician to the London Hospital, &c. &c.

8vo, cloth, price 5s.

---

### STONE'S MEDICAL PORTRAIT GALLERY.

TWENTY-ONE PORTRAITS IN LITHOGRAPHY ARE NOW OUT.

*Five Shillings each.*

| | | |
|---|---|---|
| MR. AVERY. | SIR JOHN FORBES. | MR. PAGET. |
| DR. BUDD. | DR. ED. FORBES. | MR. SIMON. |
| MR. BUSK. | MR. GAY. | MR. SKEY. |
| MR. COULSON. | MR. HANCOCK. | DR. ED. SMITH. |
| MR. SANDS COX. | MR. HOLDEN. | MR. SOUTH. |
| SIR W. ELLIS. | MR. LUKE. | DR. TODD. |
| MR. FERGUSSON. | SIR J. R. MARTIN. | MR. TRAVERS. |

No. 22, just Published, is an ENGRAVED PORTRAIT, by WALKER, of the distinguished Microscopist, the late Professor QUEKETT, F.R.S., price Half-a-Guinea; or India Paper Proofs, with his Original Autograph, One Guinea.

---

*** *All Medical and Surgical Books, Periodicals, &c., supplied as soon as published, with dispatch, correctness, and punctuality.*

